# Extraction, purification, and activity of protease from the leaves of *Moringa oleifera*

**DOI:** 10.12688/f1000research.15642.1

**Published:** 2018-07-30

**Authors:** Swarnali Banik, Shrutidhara Biswas, Srabani Karmakar

**Affiliations:** 1 Department of Biotechnology, Techno India University, Kolkata, West Bengal, 700064, India; 2 Department of Biotechnology, Indian Institute of Technology, Guwahati, Assam, 781039, India

**Keywords:** Casein, Enzyme activity, Leaf extract, Moringa Oleifera, Plant-derived proteases, Protein purification.

## Abstract

**Background**: Proteases cleave proteins, thereby providing essential amino acids for protein synthesis, and degrade misfolded and damaged proteins to maintain homeostasis. Proteases also serve as signaling molecules, therapeutic agents and find wide applications in biotechnology and pharmaceutical industry.  Plant-derived proteases are suitable for many biomedical applications due to their easy availability and activity over a wide range of pH, temperature, and substrates. *Moringa oleifera* Lam (Moringaceae) is a very common food plant with medicinal property and geographically distributed in tropical countries. Here, we isolate proteases from the leaves of *Moringa oleifera *and characterize its enzymatic activity.

**Methods**: Proteases were isolated from the aqueous leaf extract of *Moringa oleifera* by ammonium sulfate precipitation and purified by ion exchange chromatography. Subsequently, the enzyme kinetics was determined using casein as a substrate and calibrated over different pH and temperature range for maximal activity.

**Results**: We obtained purified fraction of the protease having a molecular weight of 51 kDa. We observed that for the maximal caseinolytic activity of the protease, a pH of 8 and temperature of 37ºC was found to be most effective.

**Conclusion**: The plant-derived proteolytic enzymes are finding increasing clinical and industrial applications. We could extract, purify and characterize the enzymatic activity of proteases from the leaves of *Moringa oleifera.* Further molecular characterization, substrate specificity and activity of the extracted protease are required for determining its suitability as a proteolytic enzyme for various applications.

## Introduction

All organisms contain proteases that hydrolyze peptide bonds in order to maintain systemic homeostasis and for its normal growth and development ^1, 2^. Proteases derived from plants, animals and microbes find wide industrial applications including in the leather, food, brewery and pharmaceutical industry ^2– 4^ corresponding to approximately 60% of the total worldwide enzyme sales ^5^.

*Moringa oleifera* is one of the best known medicinal plants widely distributed in the tropical regions ^6^. It contains a mixture of several hydrolytic enzymes, in which proteases are the key enzymes reported to show pharmacological activity ^7^. We attempted to investigate the protease activity of aqueous extracts of *Moringa oleifera* leaf. Here, we have isolated and purified the protease from Moringa leaves and carried out enzyme kinetics study and find that the protease exhibited optimal caseinolytic activity in alkaline pH.

## Methods

### Preparation of crude enzyme extract

Mature *Moringa Oleifera* leaves were collected from a plant located near TIU campus, Salt Lake Kolkata and crushed along with 20mM phosphate buffer (pH 7.5) and 0.1% tween 20 detergent and protease cocktail inhibitor followed by centrifugation with plastocraft table top refrigerated centrifuge machine (Rota 4RV/FM) at 10000 rpm for 10 mins at 4°C. The crude soup was mixed with 40% ammonium sulphate to obtain the protein precipitate, which was then dissolved in 20 mM tris buffer for further evaluation.

### Determination of protein content

The total protein content of the solutions at different stages of protein purification was determined by Bradford methods ^8^ using Sigma’s Bradford reagent (B6916). In this assay, a series of BSA standard solutions (0.1 – 1.2mg/ml) were used to prepare the standard curve. Bradford assay was performed by adding 1 mL of Bradford reagent to 20 μl of each standard solutions or unknown solution, and homogenized by using vortex mixer. The samples were incubated in dark conditions for 10 minutes and the absorbance was read at 595 nm.

### SDS PAGE

We performed sodium dodecyl sulfate (SDS) polyacrylamide gel electrophoresis (PAGE) using 12% resolving and 5% stacking gels for separating proteins. We followed the Laemmli’s method ^9^ for gel electrophoresis. The samples were mixed with equal volume of gel loading buffer and heated at 95°C in dry heating bath for 2 mins. The electrophoresis process was run with 90 V for first 10 mins and then run at 150 V with Biorad mini protean gel electrophoresis system. After complete run the gel was stained with Coomassie Brilliant Blue. We have used protein marker (10kD to 250 kD) from GCC biotech (Pre-stained protein marker GCR-P4B) for determination of molecular weight. We imaged the gels in Biorad gel documentation system. Acrylamide, bis acrylamide, Tris and TEMED (T9281) are from Sigma Aldrich. Coomassie Brilliant Blue R250 (93473) and Ammonium per sulphate (28575) was from SRL (Sisco Research Laboratories).

### Purification of protein

*Dialysis:* The pellet dissolved in Tris buffer as obtained above was then dialyzed in 3.5cm/ml dialysis tubing (SIGMA Aldrich D6066 overnight in a magnetic stirrer by immersing the tubing in a buffer containing Tris (pH 8) and phenylmethylsulfonyl fluoride (PMSF) SRL, which was repeated thrice for complete exchange of buffer.

*Diethylaminoethyl (DEAE) cellulose ion exchange chromatography:* The protein sample was loaded in the DEAE cellulose (SIGMA Aldrich 30477) column. Ion exchange column chromatography was carried out by using an assembly of Biorad’s Econo pump model EP-1, UV monitor and chart recorder from Atto, Japan and Biorad’s fraction collector model 2110. A gradient of 0.05 M to 0.5 M NaCl was used to elute the protein from the column. The gradient was run for 150 min with a flow rate of 1ml/min. Optical density (OD) of all the fractions were taken at 280 nm with Schimadzu 2401 UV Vis Spectrophotometer.

### Bovine serum albumin (BSA) digestion

Samples at different stages of purification were tested for albuminolytic property of protease by using BSA SIGMA as substrate. BSA digestion was performed at 37°C and pH 7.5 for 1 hour. Further, each of the samples were mixed with protein gel loading dye in 1:1 ratio and loaded in SDS PAGE and the gel was imaged with Biorad gel documentation system.

### Protease activity assay

In this assay, β-casein was used as substrate. If protease digests casein, the amino acid tyrosine is liberated along with other peptide fragments. Folin’s reagent reacts with free tyrosine to generate a blue colored product, which is quantifiable and measured as an absorbance value on the Schimadzu UV 2401 spectrophotometer at 660 nm. A tyrosine standard calibration curve is constructed to determine the amount of tyrosine released after the proteolytic activity. A series of tyrosine standard solutions at different concentrations (5 – 50 μg/mL) were prepared from the 0.18mg/mL L-tyrosine stock solution with deionized water. L-tyrosine was purchased from Himedia, Fohlin’s reagent was obtained from SRL and β-casein from SIGMA.

### Effect of pH & temperature on the protease activity

We have assayed the protease activity in terms of caseinolytic activity with plant leaf extracts at different stages of purification (crude soup is the initial supernatant after homogenization and centrifugation, 40% ammonium soup is the phosphate dissolved pellet after 40% ammonium sulphate fractionation and pooled soup is the final collection of pure fractions came from DEAE cellulose column). All the three samples were dialysed to remove protease inhibitor and EDTA before the protease assay. The protease activity of pure protein was examined at different pH range 4–9 and temperature range 4–70°C.

### Enzyme kinetics assay at different β-casein concentration

The enzyme activity assay for protease was conducted with different concentrations of β-casein as substrate, at pH-8 in 37°C respective optimum conditions as determined with the previous experiments described above (optimum temperature and pH conditions). Here the substrate concentration (β-casein) varied in the range (0.81, 1.6, 2.4, 4.03, 5.2) mg/ml keeping the enzyme concentration fixed.

## Results

*Moringa oleifera* leaves are reported to contain protease but there are no detailed studies on the purification and kinetic parameters of the enzyme. Here, we obtain partially purified protease from the aqueous extract of the leaves by ion exchange chromatography such that in anion exchange the proteins show a peak at 280 nm implying a positively charged protein.

### Purification of protease

**Figure 1.**
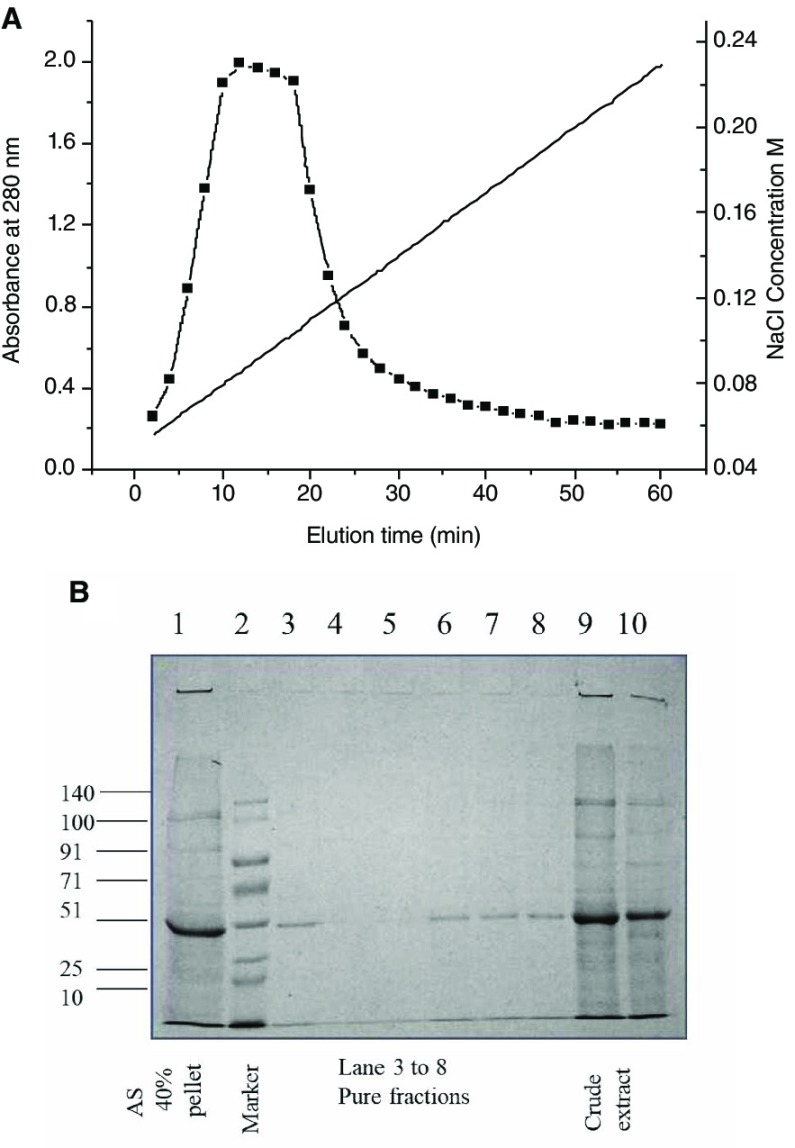
**A**. Chromatogram for the purification of protein from *Moringa oleifera* shows the elution time versus absorbance at 280 nm and the corresponding NaCl gradient profile (ranging from 0.04M to 0.25M) for maximal elution **B**. SDS PAGE of the crude extract and fractions after purification by DEAE cellulose ion exchange chromatography. Lane 1 shows extract after 40% ammonium sulphate precipitation, lane 2 shows the prestained molecular weight marker from GCC biotech marking 140, 100, 91, 71, 51, 25 and 10 kDa bands, lanes 3 to 8 represent fractions after column purification, lanes 9 and10 show the bands from crude leaf extract.

The protein concentration from mature *Moringa oleifera* leaves at various stages of purification is shown in Table 1, which was purified by DEAE cellulose ion exchange column chromatography. The chromatogram for purification is shown in Figure 1A. The purified fractions were observed in 12% SDS PAGE ( Figure 1B). The protein was of 51 kDa according to molecular weight markers.

**Table 1.** Total protein content in different stages of purification.

Samples	Protein concentration(mg/ml)
Crude	0.56
40%	0.55
Pooled	0.22

### BSA digestion and SDS PAGE

**Figure 2.**
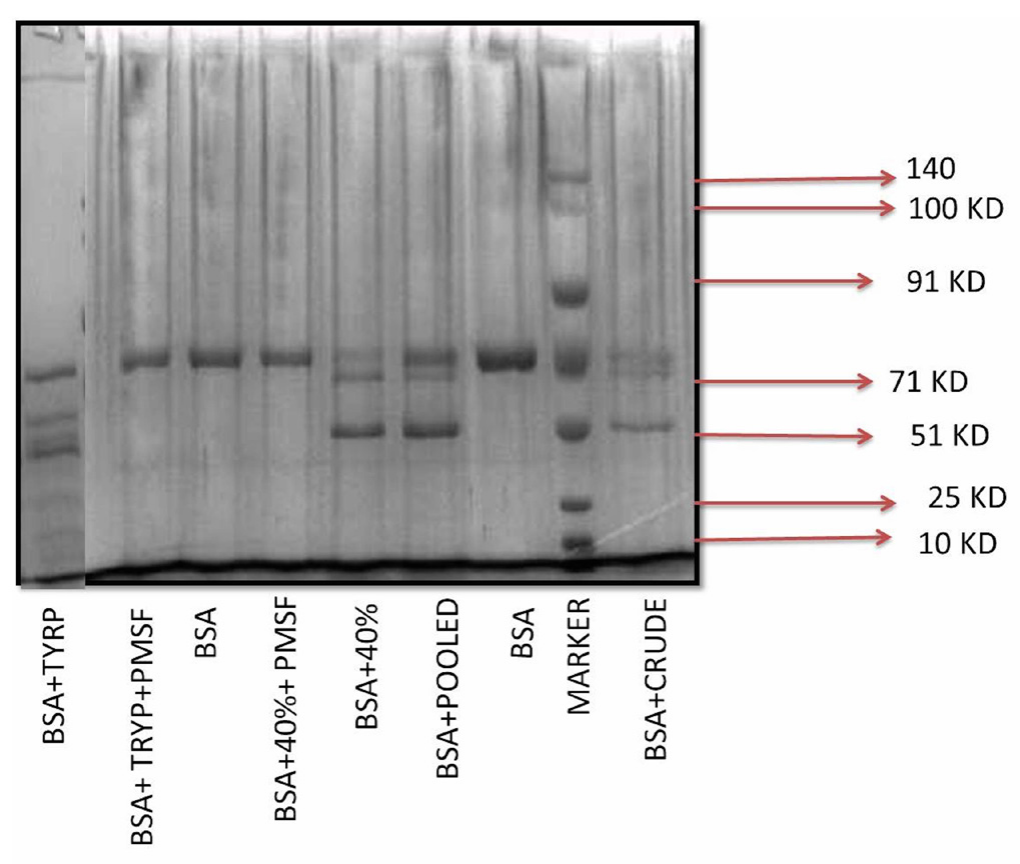
SDS PAGE showing BSA fragmentation by *Moringa oleifera* crude enzyme with trypsin as positive control. Lane 1 is BSA and trypsin, lane 2 is BSA + trypsin + PMSF, lane 3 is BSA, lane 4 is BSA +40% Ammonium Sulphate cut + PMSF, lane 5 is BSA + 40% Ammonium Sulphate cut, lane 6 is BSA + Pooled pure protein, lane 7 is BSA, lane 8 is prestained molecular weight marker from GCC biotech showing 140, 100, 91, 71, 51, 25 and 10 kDa bands and lane 9 is BSA + crude leaf extract.

Results from Figure 2 shows that both crude extract and 40% ammonium sulfate fractionated sample possesses protease activity and is able to produce fragments of BSA (lane 5, 6 and 9).

### Biophysical characterization of the protease

**Figure 3.**
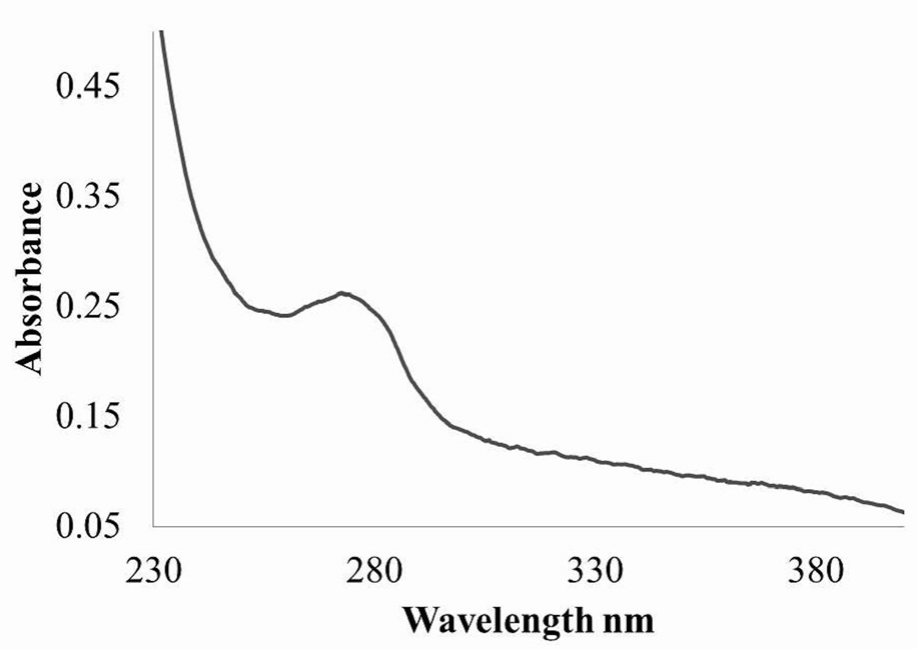
UV-Vis absorbance spectra from (230 nm to 400 nm) of the purified protein from *Moringa oleifera* leaf extract is shown.

UV-vis absorption spectra of the pure protease were shown in Figure 3. A single peak at 280 nm can be observed for the pure protein.

### Effect of pH on protease activity with β-casein as substrate

**Figure 4.**
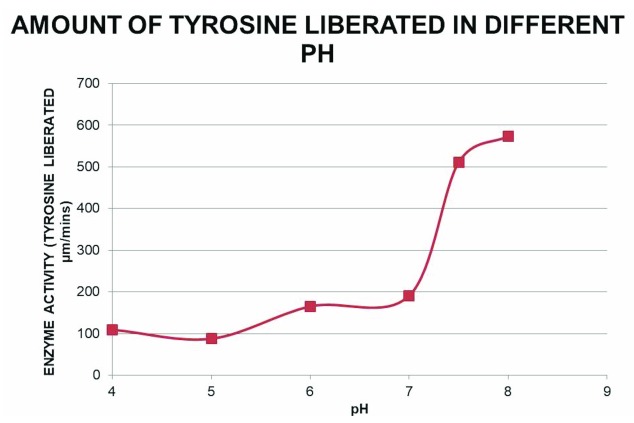
Effect of pH on the caseinolytic property of the protease. Protease activity of the pooled pure fractions on β-casein degradation is plotted against different pH (4, 5, 6, 7 and 8) at 37ºC. Free tyrosine liberated due to β-casein degradation was monitored with Folin-Ciocalteau reagent at 660 nm and the corresponding amount was measured from the tyrosine standard curve.

In both crude extract and purified protein, protease activity was measured as described in methods. Reactions in different pH 4, 5, 6, 7, 8 and 9 were done ( Figure 4). The results showed maximum activity at the pH 8.0. Therefore, the enzyme is an alkaline protease.

### Effect of temperature on protease activity with β-casein as substrate.

**Figure 5.**
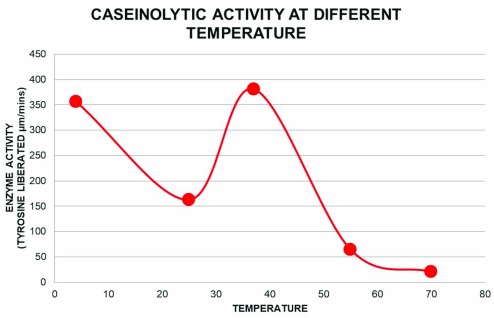
Effect of temperature on the caseinolytic property of the protease. Protease activity of the pooled pure fractions on β-casein degradation is plotted against different temperature (4, 25, 37, 55 and 70°C) at pH 8. Free tyrosine liberated due to β-casein degradation was measured as described earlier.

The protease assay with β-casein as substrate was performed at a range of temperatures; 4°C, 25°C 37°C, 55°C and 70°C ( Figure 5) according to the methods described above. The enzyme activity was found to be maximum at 37°C.

### Enzyme kinetics

**Figure 6.**
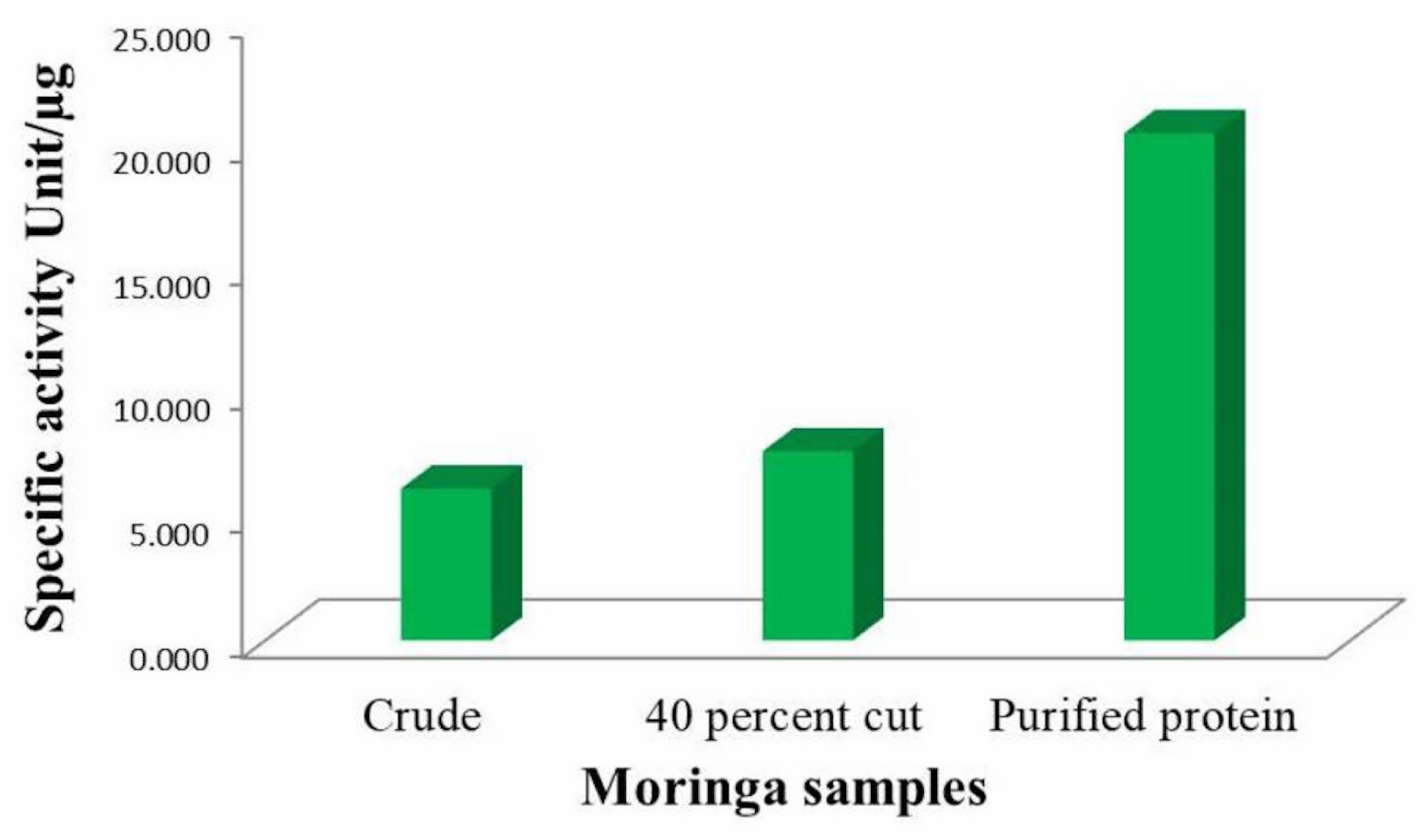
Comparison of specific activity of crude extract, 40% ammonium sulfate fraction and purified protease at optimum pH 8 and optimum temperature 37ºC is represented as a bar diagram. Free tyrosine liberated due to β-casein degradation was measured as described earlier. Enzyme activity present per amount of enzyme is calculated as specific activity of the protease.

Specific activity of the protease was calculated by enzyme activity from the protease assay using β-casein as substrate and the total protein content of the protease solution. We can see a large increase in specific activity after the final purification ( Figure 6).

**Figure 7.**
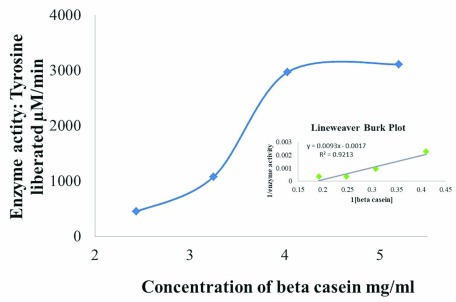
Protease activity with different β- casein concentrations is plotted to get Michaelis Menten curve and the double reciprocal plot (1/substrate concentration versus 1/ enzyme activity) is shown on the inset. K _M_ and V _max_ obtained from the double reciprocal plot are 5.47 mg/ml and 588.23 µM/min respectively.

We have seen increasing protease activity in the initial substrate concentration range and then saturation of protease activity above concentration of 4.03 mg/ml β-casein ( Figure 7). The graph as a result follows conventional Michaelis Menten kinetics. We calculated K _M_ and V _max_ from the corresponding double reciprocal plot i.e. Lineweaver Burk plot as shown in the inset graph ( Table 2). K _M_ is 5.47 mg/ml and V _max_ is 588.23 μM/min.

**Table 2.** K _M_ and Vmax from enzyme kinetics.

KM mg/ml	Vmax µM/min
5.47	588.235

Enzyme kinetics data Zip file containing underlying data of all enzyme activity assays with raw gel images Click here for additional data file. Copyright: © 2018 Banik S et al. 2018 Data associated with the article are available under the terms of the Creative Commons Zero "No rights reserved" data waiver (CC0 1.0 Public domain dedication).

## Discussion

Our study concludes that mature leaves from *Moringa oleifera* contains a protease with an approximate molecular weight of 51kD, with an optimum temperature of 37°C and optimum pH of 8.0 for its caseinolytic property. This is the first report of purification of a protease from *Moringa oleifera* to our knowledge. Further determination of molecular characterization, substrate specificity and activity of the protease are required to determine its suitability for industrial applications.

## Data availability

The data referenced by this article are under copyright with the following copyright statement: Copyright: © 2018 Banik S et al.

Data associated with the article are available under the terms of the Creative Commons Zero "No rights reserved" data waiver (CC0 1.0 Public domain dedication). 

Dataset 1: Enzyme kinetics data. Zip file containing underlying data of all enzyme activity assays with raw gel images 10.5256/f1000research.15642.d212249
