# Supplementary material for: Extraction, purification, and activity of protease from the leaves of Moringa oleifera
Source: F1000Res. 2018 Jul 30;7:1151. [Version 1] doi: 10.12688/f1000research.15642.1 (PMC6171725; doi:10.12688/f1000research.15642.1)
Supplement: Peer review history

# Peer review: Referee response for version 1

**DOI:** 10.5256/f1000research.17067.r36602

**Published:** 2018-09-25

**Authors:** Somsubhra Nath

**Affiliations:** 1 Basic Research and Molecular Biology division, Saroj Gupta Cancer Centre and Research Institute, West Bengal, India

The manuscript by Banik *et al. *describes the isolation and further characterization of protease activity of partially purified protein fraction from the leaves of *Moringa oleifera*. The manuscript is well-written with detailed description of materials, methods and results. It, indeed, identified the protease activity with sufficient detailing. It has the potential for future investigation of biochemical characterization and functional relevance of this plant derived protease.

 However, there are a few concerns, as pointed below:

1. In Figure 2, it is apparent that the first lane is not a part of original figure. Adding a separate lane in an otherwise complete picture is not accepted. A repeat experiment with all the lanes is suggested.
2. The referencing requires to be more elaborate.
3. The discussion should describe potential application of the findings, based on literature review.

Addressing these issues will strengthen the acceptability of the finding described in this manuscript.

I have read this submission. I believe that I have an appropriate level of expertise to confirm that it is of an acceptable scientific standard.

# Peer review: Referee response for version 1

**DOI:** 10.5256/f1000research.17067.r38435

**Published:** 2018-09-18

**Authors:** Sujit Roy

**Affiliations:** 1 Department of Botany, UGC Centre of Advanced Studies, The University of Burdwan, Burdwan, West Bengal, India

The article entitled ‘ Extraction, purification, and activity of protease from the leaves of *Moringa oleifera*’ by Banik et al. appears to be an interesting topic related to purification of protease from an economically and medicinally important plant species *Moringa oleifera*.

 Overall, the manuscript is well composed, the background and objectives of the study has been mentioned clearly and also justified. The introduction part also appropriately reviews some of the relevant information. The results are sound and explained properly and supported with further explanation in the Discussion part. Therefore, in summary, I’m recommending acceptance and publication of this article.   

 However, for future research interest, the introduction section could have been more extensive, indicating relevant information from recent past and current studies for getting meaningful insight into the background of this research as well as the lacunae which motivated to set the objectives for carrying out this study.

 The methods part is quite sufficient. However, for the purification part, describing the isolation of the indicated protease activity from Moringa leaf extracts may be more extensive and apart from Coomassie blue staining, quality of purification may be assessed by silver staining procedure to compare the results and enrichment of purification after the column chromatographic techniques.

 Finally, a discussion section may be included to compare and discuss the findings in light of the related research. In concluding note, application and future research possibilities using the gained knowledge and information may be mentioned.

I have read this submission. I believe that I have an appropriate level of expertise to confirm that it is of an acceptable scientific standard.

# Peer review: Referee response for version 1

**DOI:** 10.5256/f1000research.17067.r38180

**Published:** 2018-09-18

**Authors:** Rajat Banerjee, Priyanka Biswas

**Affiliations:** 1 Department of Biotechnology, Dr. B. C. Guha Centre for Genetic Engineering and Biotechnology, University of Calcutta, Kolkata, West Bengal, India; 2 Department of Biotechnology, Centre for Genetic Engineering and Biotechnology, University of Calcutta , Kolkata, West Bengal, India

The research article entitled “Extraction, purification, and activity of protease from the leaves of *Moringa* *oleifera* by Banik S, Biswas S and Karmakar S” explains the extraction, purification and activity of a protease that has been isolated from the leaves of the plant *Moringa oleifera*. The work is unique and can be of great applications since *Moringa oleifera *is reported to have various medicinal properties.

 This work may be recommended for indexing only after incorporation of a few changes listed as follows:

**Major Revision:**

1. Figure 2 – Lane numbers are missing; Marker positioning wrong; data of BSA+POOLED+PMSF and BSA+CRUDE+PMSF missing. Try to include these data for a better clarification to the readers.
2. Figure 4 – What happens after pH 8, is not reflected in the graph? Then how can this be concluded as optimum pH? A range of pH 4 – 12 at least, would be better for any such conclusion.
3. Figure 5 – The data points are very scattered. More data points must be included in the graph for proper conclusion, especially between 25°C and 37°C and 37°C and 55°C.
4. Figure 7 – This is very confusing. The graph does not look like a Michaelis – Menten graph. Number of data points must be increased. K _m_ is outside the substrate concentration range taken in the graph. K _m_ is usually expressed in units of μM or mM and not mg/ml. The Lineweaver-Burk plot is also erroneous. If the linear line is extrapolated backwards, it will give a negative y-intercept, i.e., a negative 1/V _max_ value. The V _max_ values obtained by the authors can neither be correlated to the Michaelis – Menten graph nor to the Lineweaver-Burk plot. Then how is this value obtained?

**Minor Revision:**

1. Under the Methods section, in Preparation of crude extract and Purification of protein, the authors have used “Tris” buffer. It is usually written as Tris-HCl buffer. Moreover, under the two above mentioned sub-headings, somewhere the pH of the buffer is missing and elsewhere its concentration. Clear information should be provided.
2. Under the Enzyme kinetics assay, it is written “keeping the enzyme concentration fixed”. The concentration used must be specified.
3. Figure 1B – is a bit confusing. Marker should be preferably loaded into any of the side lanes. AS 40% pellet also has multiple bands, quite similar to the ladder. If possible, change the gel picture with proper loading arrangement and labelling.
4. Figure 3 – The peak at 280 nm is quite blunt and the 260/280 ratio is close to 1, suggesting the presence of impurities. Purer fractions must be used.
5. Figure 6 – There is no need to write “Moringa samples” in the X-axis title. Instead, it should be mentioned in the figure legend as “Samples from *Moringa oleifera*”.
6. Why is this paper cited as a reference as well? Can this be done? Details about the datasheet is provided in the manuscript itself, after conclusion.

We have read this submission. We believe that we have an appropriate level of expertise to confirm that it is of an acceptable scientific standard, however we have significant reservations, as outlined above.
